# Numerous Paragraphs of General Interest to the Profession

**Published:** 1893-09

**Authors:** 


### Medical News and Miscellany.

**Dr. J. M. Gore** has recently located at Alvord.

**Dr. E. E. Johnson** has located at Ladonia, Texas.

**Dr. J. W. McLaughlin,** of Austin, has gone to the Pan-American.

**Dr. T. T. Jackson,** graduate Texas Medical College last session, has located at Iredell, Texas.

**Death of Mrs. Reuss.**—Died at her home in Cuero, Texas, Saturday, Aug. 26, Mrs. Anna Gesiene Reuss, wife of Dr. J. M. Reuss, Sr., of Cuero.

**Prof. Allen J. Smith, M. D.,** Texas Medical College, and Associate Editor TEXAS MEDICAL JOURNAL, attended the Pan-American Congress in Washington.

**A citizen** of Brooklyn has offered to undertake half the cost of a $100,000 building for the use of the various medical societies of that city, provided these societies will undertake the other half.—*Ex.*

**Wanted.**—Two copies each of the January and March numbers of *Daniel's Texas Medical Journal*, this series,—1893. A credit on subscription will be given if subscriber, or 25 cents in cash if not.

**The** Belgian Academy of Medicine has offered a prize of 4,000 francs ($800) for the best essay upon the pathology and treatment of epilepsy. Papers in competition must be presented before February 1, 1894.—*Ex.*

**Dr. M. H. Oliver,** of Ennis, Texas, one of the oldest members of the Texas State Medical Association, and for years known as the guardian of its portals as member of the Judicial Council, died suddenly at his home on the 5th inst.

---

**Dr. Frances Van Gasken,** a graduate ot the Women's Medical College of Pennsylvania, has received the appointment of Assistant Health Inspector of Philadelphia. Dr. Van Gasken's old home is Luling, Texas, where her brother is practicing medicine and looking after the aqueous interests of the inhabitants.

---

**Death of Mrs. Ellison.**—Our friend, Dr. W. A. Ellison, of Manchaca, this county, had the misfortune to lose his wife on August 1st, ult. She died after a brief illness. She leaves three young children,—girls, aged seven, ten and twelve. The JOURNAL extends its cordial sympathy to the doctor in his bereavement.

---

**Died.**—In Cleburne, Texas, August 2, ult., Tiny Marguerite Wagley, daughter and only child of Doctor and Mrs. T. J. Wagley. She was born in Berlin, Germany, July 15, 1890, and was therefore just a little more than three years of age. The TEXAS MEDICAL JOURNAL extends sincerest sympathy to the bereaved parents.

---

**Dr. T. J. Tyner** and Mrs. Tyner left Austin Aug. 30 for Waukesha, where they spent a few days before going to Washington. Dr. Tyner will read a paper at the Pan-American Medical Congress and will, after adjournment of Congress, spend a month in traveling and resting, preparatory to resuming his practice at Austin.

---

**Dr. H. W. Dudley,** of Hillsboro, Texas, so well and favorably known throughout Texas as one of our ablest physicians, has removed to Hot Springs, Arkansas, in order to have a wider field. Dr. Dudley is a close student, and an ethical physician of twenty years experience. He for one will uphold "regular medicine" in the hot bed of quackery.

---

**In the Quiz Class.**—Prof. of Hygiene and State Med.—"What do you understand, Mr. Sporrer, by 'preventive medicine'?—give me an illustration."

Mr. Uppie T. Sporrer, of Texas.—"P-p-pr'ventive med'cin is —*er-rer*—med'cin what per-vents; like w-w-when un gives lead 'n opium f-f-fur the dier-ree! See?"

**Dr. M. D. Sterrett,** of Beekville, Panola county, has just returned from a visit to his old home, in Mississippi, where he went, he says, for health and pleasure. The JOURNAL hopes he had, and will continue to have, a large share of both. We do not know a harder worked and working disciple of Æsculapius anywhere, or one who better deserves a rest, and "health and happiness."

---

**Well,** the great "International Health Conference" will take place at Chicago, 9th to 15th of October, proximo. It will be a joint session of the American Public Health Association and the Section on Hygiene and Public Health of the World's Congress Auxiliary. Dr. Swearingen will read a paper there which will "wake the natives" of our sister Republic to a realizing sense of their remissness in sanitary matters.

---

**The** Medico-Chirurgical Hospital, attached to the Medico-Chirurgical College of Philadelphia, received a grant of $100,000 from the last legislature of Pennsylvania. To the faculty of this, the youngest of the Philadelphia medical colleges, have been elected Dr. Seneca Egbert, to the chair of Hygiene, and Dr. J. Madison Taylor, to the chair of Diseases of Children. Both these gentlemen are graduates of the University of Pennsylvania.

---

**Errata.**—In our last issue an error occurred in the number of graduates at two of the medical colleges. The Medical Department, University of Tennessee (Nashwille Medical College), was put down at "14" when it should have been 114; and the number graduated from the Vanderbilt and University of Nashville was put down at "150" when it should have been 138. This is an unfortunate occurrence, one which we greatly regret. See mention elsewhere.

---

**A Moderate Drinker.** — When we examined Fritz for the Knights (Fritz is a big burley German who delivers Lemp's beer around town in kegs), we asked, "Do you drink habitually?" "Yes, beer." "Are you a moderate drinker or do you ever drink to excess?" "Oh, no, I drinks very little beer sometimes already." "About how much a day?" we asked. "Oh," said Fritz, "I don't know (reflecting a moment with his head down, evidently summing up the drinks he takes on his rounds with his customers), "It been about *forty schooners*, may be; I don't done drink more as dat any more."

**Dr. T. D. Wooten,** President of the Board of Regents of the Texas University, returned on the 1st instant from his summer vacation in Colorado, much recuperated in strength. He will leave Austin on the 18th instant, with his family, for Chicago, thence to New York, where his sons, Joseph Wooten and Goodall Wooten, will matriculate at the College of Physicians and Surgeons for their third and last course of lectures, having attended two courses at the Texas Medical College (Medical Department University of Texas), at Galveston.

---

**Tell the Doctor** that if he is not a subscriber to the TEXAS MEDICAL JOURNAL he is missing a real heap of good things useful in his business or otherwise; it is great. If he does not subscribe he will not have good luck this year. All subscriptions sent in *now*, and up to December 1st with the cash, will be dated January 1, 1894, the issues from now to January being *thrown in*; lagniape. The price asked, $2, is so small, that once paid it will not be felt, sixteen cents a month; the price of *one cigar* every ten days, or three "beers" a month will pay it.

---

**Dr. S. H. Stout,** so long and so favorably known to the Texas profession, has removed from Cisco to Dallas. It will be remembered that Cisco was destroyed by a cyclone, and while Dr. Stout's residence was not destroyed, and his losses were slight, compared to others, his occupation was taken away, the town never recouped and, of course, practice was no longer remunerative. The JOURNAL commends Dr. Stout to the people of Dallas —the profession all know and appreciate his worth—as a physician of the highest order of ability, and a gentleman of true worth, deserving in every way of the most implicit confidence and esteem.

---

**Two** deaths from cholera occurred at Jersey City August 29th, officially reported by the Marine Hospital Surgeon at that point, created considerable excitement, but it has died out completely. Science is too much for cholera and yellow fever in America. Yellow fever tried to get in its work at three points this year. First, two deaths at Pensacola were reported; then Dr. Brenham, of the M. H. S., died at Brunswick, Ga.; later a "case" on the dock at Tampa, Fla., was telegraphed to all health officers, but the "case" turned out to be a false alarm. It is strange the M. H. S. will send a young and inexperienced surgeon like Dr. Brenham into yellow fever, when they have so many veterans available, who being "acclimated," run no risk.

**Prof. James Kennedy, M. D., Ph. G.**—The Board of Regents University of Texas, held a meeting in Austin, September 4th, for the transaction of general business pertaining the opening of the session of the University, and to select a Professor of Pharmacy for that chair, recently created in the Faculty of the Medical Department. Dr. James Kennedy, of San Antonio, was chosen, there being, we are informed, fifty odd applications; one from a gentleman who has been Lecturer on Pharmacy in a Baltimore Medical College. The election of Dr. Kennedy is a high compliment, worthily bestowed. We doubt if he has a superior as a Pharmacist anywhere, and having an easy flow of language and an impressive manner, he will make a good teacher.

---

**Death of Dr. Styles, Jr.**—Died at his residence in Stephensville, Texas, July 16th, 1893, Thomas Wright Styles, aged thirty five years and one week. Dr. Styles was born in Laurens District, S. C., where his ancestors had lived from the earliest history of the State, and where his grandfather, Gen. Thomas Wright, was an influential citizen.

In 1866, his father, Dr. Samuel Farrow Styles, moved his family to Washington county, Texas, where they have resided up to the present time.

In 1878, Wright Styles graduated from Baylor University, Texas, with distinguished honors. He then prosecuted the study of medicine with his father until his matriculation in Tulane Medical University in New Orleans, whence he graduated with distinction in 1882.

---

**The Old Man Was All Right.**—You know an Englishman can not see a joke. When Mark Twain's inimitable mock lament over the tomb of Adams was read to an English lord, as a fine specimen of American humor, the Britisher didn't "crack a smile," but looking as sober as a judge said, that was not humor, it was a lie. Dr. C. U. Later tells us of his trying to play off an old "gag" on an Englishman, a physician of his acqaintance. The Englishman had an obstetrical case—tedious labor—in which both mother and child were lost. Dr. Later said, "Well, I hope you'll be able to save the old man at least." Whereupon the other looked at him in wide-eyed astonishment and said, "Why, there's nothing the matter with the old man; the old man is all right." Later went out and kicked himself.

---

**Surgical Instruments.**—A recent number of Meyer Bros. & Co.'s St. Louis *Drug Magazine* states that the Fort Worth Pharmacy Company, of Fort Worth, carries the largest stock of Surgical Instruments and mechanical curative devices in the Southwest. We are personally acquainted with these people and have a correct knowledge of their stock, fully endorse the above, and will further say, their prices we know to be as low as any of the Eastern houses, and that they are now giving good satisfaction to five hundred Texas doctors whom they number as their patrons. A year ago we asked the profession to aid us in building up this house—a home institution—where orders could be promptly filled and delivered. We have now to say that satisfactory results are being obtained. The Fort Worth Pharmacy Company are also agents for three manufactories of electric Batteries and two manufactories of Physicians' Chairs. Write to them for what you may want.

---

**Dr. T. J. Bennett,** Managing Editor and Publisher of the *Texas Sanitarian*, left Austin September 1st, for Washington, where he will attend, as delegate from the Texas State Medical Association, the Pan-American Medical Congress. Thence Dr. Bennett will go to Chicago, to see the sights, take in the hospitals, etc., spending two weeks there; thence he will go to New York and study medicine and surgery in the polyclinic and post-graduate colleges and the big hospitals. While in Washington Dr. Bennett will, of course, attend the meeting of Medical Editors, and will also attend and assist in organizing the Medical Publishers' Association, representing his own journal, the *Texas Sanitarian*, and by proxy, the TEXAS MEDICAL JOURNAL. The JOURNAL commends its colleague and contemporary to the brethren everywhere, as a gentleman in every way worthy of the highest esteem and confidence, and as a physician of eminent ability and professional standing.

---

**Vnet.**—It is well known that "animal spirits" find vent in overt acts,—they must be "worked off." The capers and frolics of young animals illustrate what we mean. A boy, for instance (as a type of young animal), would *burst* if he were compelled to sit still,—be perfectly quiet for any length of time; he is bound to give expression in acts to the exuberance of vital force animating him. We all remember the little fellow who got "awfully rested" at church. Likewise, one's "feelin's" must have vent also, sometimes. This takes the form of cuss words with the—indiscreet; with others, in slamming doors, stamping the feet; with some women, for instance, relief is found in tears. But,—there are some states which, so far as we have tried them, none of these remedies will relieve. The chestnut about the old fellow who, when asked why he didn't cuss, replied he couldn't do justice to the occasion, will be recalled.

Well—our printer;—he has made us say "old ancient" (see "Function of Pharmacist" in our August number), just as if there were or could be any *new* ancient or *young* ancient. We will take our affidavit that it was not in copy nor in proof. Just where he got "old"—unless out of his head, as Jack did the fiddle, and had wood enough for another—we don't know. Saw it only after JOURNAL was bound.

What would have been the use of killing the printer, or of cussin'? Neither one would have kept the *New York Medical Record* from twitting us about it. Well, let 'em. Smart Alecks always notice such little mishaps, and pretend to think our editor doesn't know any better. But in the same issue there was a typographical error much more exasperating: it would justify "cussin'" and killing the whole printing establishment. It was in giving the graduates at the several Southern medical colleges —taken (reprint) from an exchange;—the printer, not we, only credited 14 to the Medical Department of the University Tennessee, when it should have been 114. We make our sincerest apologies, and if Bro. Eve says so, we will go at once and kill the printer. There, we feel better. We have worked off some of our indignation, but can't do full justice to the subject.

---

### PHYSICIAN'S CHAIRS—A WONDERFUL OFFER.

For a limited time only, we offer a *$65 Surgical and Gynecological Chair* for $37.50, cash, or $45 on monthly payments, viz.: $20 cash with order, and $5 monthly.

This new and wonderful chair has been viewed with wonder and admiration, and is now used by many of the foremost and most noted surgeons and gynecologists in the United States. In its construction it combines the desirable points of all the best chairs, and all objectional features which are so characteristic of chairs generally have been most artfully overcome. Not a single tenable objection can be offered against this "*New Indianapolis*" chair. It is *strong* and *durable* (covered with leather, lasts a life-time). It is *ornamental* and *beautiful*. It is the *easiest* chair ever made to *manipulate*. In every *contest* for *points* it *annihilates competition*. Every position desired is *instantly* and *easily* obtained without muscular effort. It is the most simply constructed chair on the market, being absolutely free from complications and triggers, and is warranted to give *perfect* satisfaction. Send your orders at once, to DRAKE & WOOD CO. (Manufacturers), Austin, Texas.

